# An Automatic and Novel SAR Image Registration Algorithm: A Case Study of the Chinese GF-3 Satellite

**DOI:** 10.3390/s18020672

**Published:** 2018-02-24

**Authors:** Yuming Xiang, Feng Wang, Hongjian You

**Affiliations:** 1 Key Laboratory of Technology in Geo-spatial Information Processing and Application System, Institute of Electronics, Chinese Academy of Sciences, Beijing 100190, China; wfeng_gucas@126.com (F.W.); hjyou@mail.ie.ac.cn (H.Y.); 2 University of Chinese Academy of Sciences, Beijing 100049, China

**Keywords:** SAR, image registration, SAR-SIFT, phase congruency, GF-3 satellite

## Abstract

The Chinese GF-3 satellite launched in August 2016 is a Synthetic Aperture Radar (SAR) satellite that has the largest number of imaging modes in the world. It achieves a free switch in the spotlight, stripmap, scanSAR, wave, global observation and other imaging modes. In order to further utilize GF-3 SAR images, an automatic and fast image registration procedure needs to be done. In this paper, we propose a novel image registration technique for GF-3 images of different imaging modes. The proposed algorithm consists of two stages: coarse registration and fine registration. In the first stage, we combine an adaptive sampling method with the SAR-SIFT algorithm to efficiently eliminate obvious translation, rotation and scale differences between the reference and sensed images. In the second stage, uniformly-distributed control points are extracted, then the fast normalized cross-correlation of an improved phase congruency model is utilized as a new similarity metric to match the reference image and the coarse-registered image in a local search region. Moreover, a selection strategy is used to remove outliers. Experimental results on several GF-3 SAR images of different imaging modes show that the proposed algorithm gives a robust, efficient and precise registration performance, compared with other state-of-the-art algorithms for SAR image registration.

## 1. Introduction

GF-3 is the Chinese SAR satellite with scientific and commercial applications, launched in August 2016. It employs a multi-polarized C-band SAR at meter-level resolution based on active phased array technology, which allows operation in stripmap, scanSAR and sliding spotlight modes for both right looking and left looking [1]. It is mainly used in the fields of oceanography, disaster reduction, water conservancy, meteorology, and so on [2]. Being the process of aligning two images of the same area, image registration becomes increasingly important due to the extensive use of SAR images. However, affected by speckle noise and different imaging conditions, the intensity and geometric information of the same ground scene in SAR images differ widely. Consequently, it is challenging for most of the existing methods to achieve an efficient and accurate registration performance for SAR images from different imaging modes.

The existing registration algorithms of SAR images can be roughly divided into two categories: area-based and feature-based. Feature-based techniques depend on detecting and matching distinctive features from the images, while area-based techniques implement the geometric transformation estimation by optimizing a similarity measure between the two images [3].

Feature-based methods mainly consist of three steps. First, significant features such as points, lines, regions and edges are extracted from the two images [4]. Second, each feature from the reference image is matched with the corresponding feature in the sensed image by various feature descriptors, such as the famous SIFT descriptor [5], shape context [6], phase congruency [7] and local self-similarity descriptor [8]. Third, the geometric transformation model between images is established using the reliable feature correspondences. Among the feature-based methods, the SIFT-like algorithms are the most widely-used techniques in SAR image registration due to the efficient performance and invariance to scale, rotation and illumination changes [9]. However, the traditional SIFT algorithm does not perform well on the SAR images due to the effect of strong speckle noise. Several algorithms have been proposed to improve the SIFT algorithm for SAR image registration. Schwind et al. [10] ameliorated SIFT by skipping of the first scale-space octave (SIFT-OCT). Fan et al. [11] improved the matching performance by skipping the dominant orientation assignment when the matching images do not have rotation transformation. By replacing the Gaussian filter with several anisotropic filters, the Bilateral Filter SIFT (BFSIFT), Adapted Anisotropic Gaussian SIFT (AAG-SIFT) and Nonlinear Diffusion Scale Space-SIFT (NDSS-SIFT) are proposed to improve the matching performance [12,13,14]. Dellinger et al. [9] proposed the SAR-SIFT algorithm specifically dedicated to SAR images by utilizing the Ratio Of the Exponentially-Weighted Averages (ROEWA) instead of a differential to calculate a gradient. Wang et al. [15] improved the SAR-SIFT algorithm by the strategies of optimal feature selection based on a Voronoi diagram and feature scale-space proportional extraction.

Although the feature-based algorithms have been widely used in the SAR image registration, there still exist several problems when applied to SAR images of different imaging modes. First, it is difficult to obtain enough correct correspondences using SIFT-like methods alone due to the differences between SAR images of different imaging modes [8]. The computational cost for constructing descriptors and searching for the correspondences is very high when the amount of feature points is large. Moreover, intensity and geometric information, such as buildings and water areas, may differ widely because of the different imaging conditions and speckle noise [16]. Consequently, the global transformation estimation that depends heavily on the selection of appropriate feature points often results in low registration accuracy [17,18].

Different from feature-based methods, area-based methods first define a template in the sensed image, then search for the optimal correspondence in the reference image using different kinds of similarity measures, including mutual information [19], the normalized cross-correlation coefficient [20] and cross-cumulative residual entropy [21]. Compared with the feature-based methods, the area-based method can robustly register multimodal images with intensity difference. Especially, Normalized Cross-Correlation (NCC) has been widely applied for image registration because of its invariance to linear intensity changes, and Mutual Information (MI) is robust to non-linear intensity differences and has been successfully applied in the registration of multispectral or multisensor remote sensing images [3]. However, these methods may lead to local extrema and high computational load [22]. Moreover, these methods may be ineffective when applied to large remote-sensing images, which are usually influenced by noise and distortions.

Recently, some new registration approaches were proposed by integrating the advantages of both the feature-based method and the area-based method. These approaches usually have a two-stage process that consists of the coarse registration and the fine registration. Liang et al. [18] presented a two-phase integrated method to overcome the respective weaknesses of registration accuracy and computational load. Phase-1 only considers the Spatial Information (SI) of feature points and finds some low-accuracy solutions. Phase-2 utilizes both SI and mutual information of intensity to search for a more accurate solution. Han et al. [23] combined the mutual information and linear features to co-register Very High-Resolution (VHR) optical and SAR images. The global translation difference between optical and SAR images is minimized from coarsest to finest pyramid images. Gong et al. [24] constructed a novel coarse-to-fine scheme for remote sensing image registration. The coarse registration process is implemented by the SIFT approach equipped with a reliable outlier removal procedure. The fine-tuning process is implemented by the maximization of mutual information using a robust search strategy in a multiresolution framework. Ye et al. [8] proposed an automatic registration method for multispectral images by integrating a scale restriction SIFT-like algorithm and a new similarity metric based on the local self-similarity descriptor in a coarse-to-fine registration scheme.

Even though these approaches have achieved robust and accurate results on various remote sensing images taken in different situations, they still have some limitations. First, the aforementioned two-stage approaches have not seriously considered the intrinsic nature of strong speckle in SAR images. Consequently, the registration performance may be poor when the SAR images to be registered are strongly corrupted by the speckle. Moreover, these approaches can be computationally expensive when solving registration of large SAR images [17]. For practical applications, it is required to have a real-time procedure for SAR image registration.

In this paper, we propose an automatic and efficient registration algorithm for SAR images of different imaging modes. The proposed approach also consists of two stages, the coarse registration and the fine registration. First, we introduce an adaptive sampling method. By integrating the method and the SAR-SIFT algorithm, two large SAR images are efficiently coarse-registered. Then, obvious translation, rotation and scale differences are eliminated to further process. In the fine registration stage, we utilize the fast normalized cross-correlation of an improved phase congruency model as a new similarity measure to find corresponding points in a local search region. In order to achieve a stable and robust performance, uniform control points are extracted and a selection strategy is used to remove outliers. The main contributions of this paper are given as follows:

1. A new coarse to fine automatic registration scheme is designed. Both the SAR-SIFT algorithm used in the coarse registration and the improved phase congruency model used in the fine registration are robust to speckle in SAR images. The fast NCC of the improved phase congruency is used as a new similarity measure in the fine registration.
2. An adaptive sampling method used in the coarse registration is proposed, which decreases the computational cost while maintaining the matching performance. A correspondence selection strategy is introduced to remove outliers.
3. To our best knowledge, this is the first paper to introduce an automatic, robust and efficient registration algorithm for the Chinese GF-3 SAR images of different imaging modes.

The rest of this paper is organized as follows. Section 2 introduces the Chinese GF-3 satellite and presents the existing problems for matching SAR images from different imaging modes. The proposed registration algorithm is described in Section 3. Experimental results on several pairs of GF-3 SAR images are illustrated in Section 4. We discuss the registration performances of the proposed algorithm in Section 5, compared with other state-of-the-art algorithms. The conclusions are drawn in Section 6.

## 2. The Chinese GF3 Satellite

The GaoFen (GF) series is the space-based system that is part of the China High-Resolution Earth Observation System, which is established as a Chinese National Science and Technology Major Project [25]. GF-3 is a multi-polarized C-band SAR satellite of the GF series, launched in August 2016. It has the largest number of imaging modes among the SAR satellites in the world, covering the traditional stripmap and scanSAR modes, as well as the wave mode and the global observation mode for ocean application. Combining the advantages of high spatial resolution with the large imaging width, the GF-3 satellite can meet the needs of different users. In addition, the GF-3 satellite is the first low-range remote sensing satellite with a design life of eight years in China, which can provide users with long-term stable data support services.

**Figure 1:**
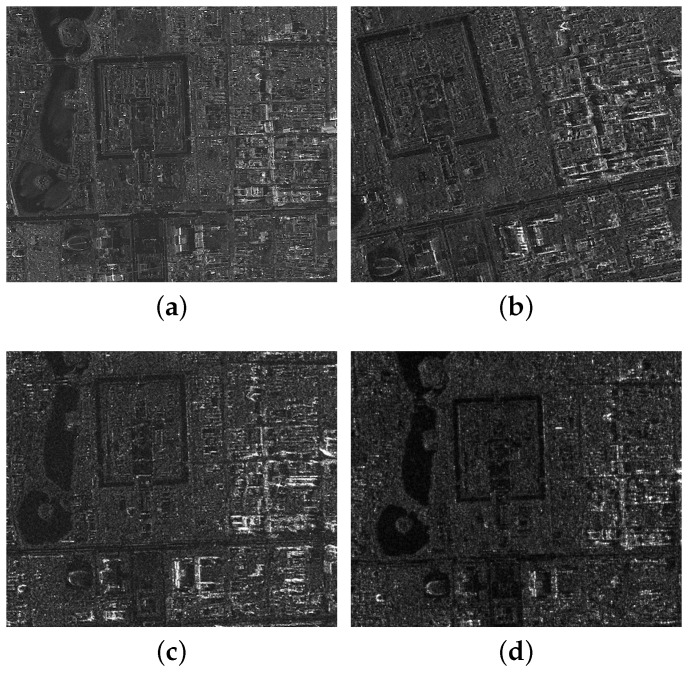
Four GF-3 images of different imaging modes. (**a**) Spotlight (SL); (**b**) Ultra-Fine Stripmap (UFSM); (**c**) Fine Stripmap One (FSMI; (**d**) Quadrupolarization Stripmap One (QPSMI).

The GF-3 satellite can monitor the global ocean and land resources in all-weather and all-day. Consequently, the GF-3 SAR images have been widely applied in numerous applications, such as disaster risk forecasting, water resources assessment and management, disaster weather, climate change forecast, and so on. Being the fundamental task of these image applications, SAR image registration needs to be accurate and efficient, which is still a challenging problem. Herein, we choose four GF-3 images to demonstrate the existing problems. Four different SAR images of the same ground scene are presented in Figure 1, where the first was imaged by the Spotlight (SL) mode in December 2016, the second imaged by the Ultra-Fine Stripmap (UFSM) mode in September 2016, the third imaged by the Fine Stripmap One (FSMI) mode in August 2016 and the fourth imaged by the Quadrupolarization Stripmap One (QPSMI) in September 2016. Since the resolutions of the four modes are different, we resize the four images to make a straightforward illustration.

It is clear that there exist differences between the four images. The water areas are changed according to the weather and wave pattern, shown in Figure 1a, and the urban areas differ widely due to the geometric distortions caused by different imaging situations. Especially for the SL mode, the high spatial resolution image further widens the existing gap of geometry information [23]. Moreover, due to the high spatial resolution, processing large volumes of remote sensing data has become a normal daily activity. The computational cost directly depends on the data volume, making the real-time registration a challenging task.

## 3. Methodology

**Figure 2:**
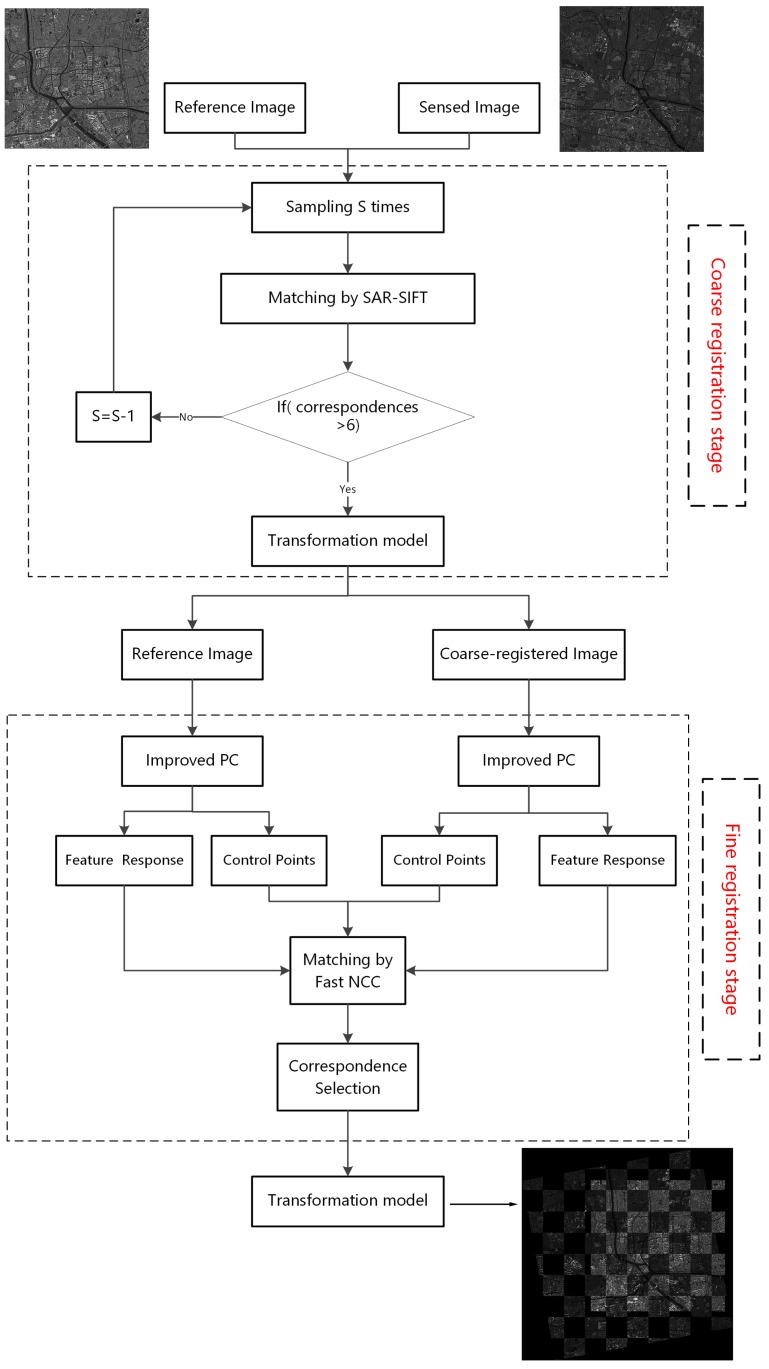
The flowchart of the proposed algorithm. PC, Phase Congruency; NCC, Normalized Cross-Correlation.

Despite being sensitive to intensity differences and speckle noise, the SIFT-like methods are still useful for initial registration to remove the obvious scale and rotation differences. Meanwhile, the area-based methods like NCC can achieve sub-pixel accuracy when the geometric distortion is small. Combining the advantages of the SAR-SIFT algorithm and the fast NCC method, a robust and efficient coarse-to-fine registration scheme is proposed. The proposed algorithm involves a two-stage process, namely coarse registration and fine registration. In the coarse registration stage, by integrating an adaptive sampling method and the SAR-SIFT, corresponding feature points are extracted. Then, the sensed image is rectified through an affine transform, named the coarse-registered image. In the fine registration stage, uniform control points are first extracted in the reference image. Then, an improved phase congruency model is proposed to extract feature responses for the reference image and the coarse-registered image, and we utilize the fast NCC of feature responses as a new similarity measure. Correspondences are obtained by finding extrema in a local search region that relate to the sampling parameter and error range in the first stage. A correspondence selection strategy is introduced to remove outliers. Finally, the coarse-registered image can be rectified using an affine transform. The flowchart of the whole process is illustrated in Figure 2.

### 3.1. Coarse Registration

The SIFT-like algorithms have shown good performance for multi-angle SAR image registration [10]. Among them, the SAR-SIFT is the sate-of-the-art method, which contains three major steps: keypoints detection, orientation assignment and descriptor extraction, keypoints matching. First, instead of constructing the Gaussian image pyramid, the algorithm starts with constructing a Harris scale space. Replacing the original gradient with the logarithmic ROEWA operator [26], the gradient by ratio is given as follows:

(1) $$\begin{array}{c} G_{h,\alpha }\left(x,y\right)=\log\left(\frac{\sum_{i=-M/2}^{M/2}\sum_{j=1}^{N/2}I\left(x+i,y+j\right)e^{-\frac{\left|i\right|+\left|j\right|}{\alpha }}}{\sum_{i=-M/2}^{M/2}\sum_{j=-N/2}^{-1}I\left(x+i,y+j\right)e^{-\frac{\left|i\right|+\left|j\right|}{\alpha }}}\right)\\ G_{v,\alpha }\left(x,y\right)=\log\left(\frac{\sum_{i=1}^{M/2}\sum_{j=-N/2}^{N/2}I\left(x+i,y+j\right)e^{-\frac{\left|i\right|+\left|j\right|}{\alpha }}}{\sum_{i=-M/2}^{-1}\sum_{j=-N/2}^{N/2}I\left(x+i,y+j\right)e^{-\frac{\left|i\right|+\left|j\right|}{\alpha }}}\right), \end{array}$$

where *M* and *N* denote the size of the neighborhood, (x,y) stands for the pixel location, α is the exponential weight parameter and *I* represents the pixel intensity. Then, the SAR-Harris function is computed at different scales, resulting in the Harris scale space. Local extrema in the scale space are selected as keypoints candidates. Second, dominant orientation is assigned to each keypoint to maintain the rotation invariance, which corresponds to the highest peak in the scale-dependent gradient orientation histogram. A circular neighborhood (size of 6σ) and log-polar sectors are employed to construct the descriptor. Third, the keypoint matching stage is similar to the SIFT algorithm, which refers to the Nearest Neighbor Distance Ratio (NNDR) method. More details about the SAR-SIFT can be found in [9].

In the coarse registration stage, we combine an adaptive sampling method with the SAR-SIFT algorithm (AsSAR-SIFT). The adaptive sampling method is described as follows: Assuming the sizes of the reference and sensed images are $M_{1}\times N_{1}$ and $M_{2}\times N_{2}$, respectively, we first down-sample the two images by *s* times to achieve MMss<500,NNss<500, where *M* and *N* are the size of the smaller image. Then, the SAR-SIFT matching is performed between the down-sampled images. After filtering by robust outlier removal, such as RANdom SAmple Consensus (RANSAC) [27], if the number of remaining correspondences is higher than a threshold $N_{t}$, the transformation model is estimated using the corresponding keypoints. Otherwise, the process returns to the beginning, and we down-sample the two images by s−1 times. The process continues until there exist enough correspondences to estimate the transformation model. Finally, the sensed image is rectified by the affine transform.

At a coarse spatial resolution, the geometric differences between the reference image and the sensed image are reduced. The reason is that each pixel is related to the density of neighborhood structures much more than their actual spatial pattern, which is not visible at a coarse resolution [28]. Additionally, the down-sampling method can be regarded as a multi-look process, which can decrease the speckle noise. Moreover, the computational cost of the coarse registration stage is significantly reduced due to the smaller image size.

### 3.2. Fine Registration

We have eliminated the obvious scale, rotation and translation differences between the reference image and the sensed image in the coarse registration stage. The SAR-SIFT algorithm has been proven to achieve a pixel-level root mean square error (RMSE) for multi-angle SAR images, 2 pixels specifically [9]. Compared with the multi-angle SAR images, the situations of SAR images of different imaging modes are more complex, so we roughly set the localization deviation as 5 pixels, then a local search region can be set as $\left[-5\cdot s,5\cdot s\right]$, both in the x-axis and y-axis directions, where *s* is the sampling times in the coarse registration stage. In the fine registration stage, uniformly-distributed Control points (Cps) are extracted using the feature responses calculated by an improved phase congruency model. Normally, a detector that uses a fixed threshold to extract keypoints from an image may lead to unevenly-distributed results [8], and keypoints are easily gathered in areas rich in structural information, such as building areas. However, the building areas differ widely under different views and resolutions, which may cause misregistrations. In order to solve the problem, we first partition the reference and coarse-registered images into several non-overlapped blocks, then an improved phase congruency model is implemented on each block to calculate feature responses. For each block, ranking the responses in descending order, we choose the highest *k* points as the control points in each block. Afterwards, the fast NCC of feature responses is utilized as a new similarity measure. Templates are first selected for each Cp in the reference image, then corresponding points are obtained by finding the maximum in the local search region using the new similarity measure. Outliers are removed by a selection strategy. Finally, the coarse-registered image can be rectified using an affine transform.

#### 3.2.1. An Improved Phase Congruency Model

As mentioned in the Introduction, there exist many similarity metrics in area-based methods, such as the Sum of Squared Differences (SSD), NCC, MI, the Local Self-Similarity (LSS) and the Histogram of Orientated Phase Congruency (HOPC) [29]. However, these similarity metrics give poor performance when they are directly applied to SAR image registration. The main reasons are the affect of speckle noise and geometric distortions in SAR images. Aiming at solving these problems, we propose an improved phase congruence model, which is not only robust to speckle noise and intensity differences, but is also efficient to represent structural information.

First, we briefly review the conventional phase congruency model [30]. Different from extracting features with the classical gradient-based operators, features can be perceived at points where the Fourier components are maximally in phase. Venkatesh and Owens showed in [31] that the local energy model is directly proportional to phase congruency. Hence, peaks in local energy correspond to peaks in phase congruency. The measure of local energy is calculated by convolving the image with a filter bank of quadrature filters, which is given as follows:

(2) $$\begin{array}{c} LE\left(x,y\right)=\sqrt{E{\left(x,y\right)}^{2}+O{\left(x,y\right)}^{2}};\;\\ E\left(x,y\right)=\sum_{n}I\left(x,y\right)*M_{n}^{e},\;\\ O\left(x,y\right)=\sum_{n}I\left(x,y\right)*M_{n}^{o}\;, \end{array}$$

where *n* represents the number of scales, *I* represents the image intensity, $M_{n}^{e}$ and $M_{n}^{o}$ denote the even and odd symmetric filters at scale *n*, E(x) represents the convolution of the image with the even symmetric filter and O(x) represents the convolution of the image with the odd symmetric filter. In order to extend the local energy model to two dimensions, Kovesi improved the model by using the log Gabor wavelets to calculate local energy over scales and orientations [30]. Then, considering the noise and blur of images, the phase congruency model proposed by Kovesi is defined as:

(3) $$PC\left(x,y\right)=\frac{\sum_{o}W_{o}\left(x,y\right)\left\lfloor LE_{o}\left(x,y\right)-T_{o}\right\rfloor }{\sum_{o}\sum_{n}A_{no}+\varepsilon }\;,$$

where *n* represents the number of scales, *o* stands for the number of orientations, $W_{o}$ represents the weighting function at the *o*-th orientation, $LE_{o}$ is the local energy model at the *o*-th orientation, $T_{o}$ is the noise level at the *o*-th orientation, $A_{no}$ is the amplitude at scale *n* and orientation *o*, given as $A_{no}=\sqrt{E_{no}{\left(x,y\right)}^{2}+O_{no}{\left(x,y\right)}^{2}}$, and ε is a small positive value to prevent the expression from becoming unstable when $A_{no}$ becomes very small.

Due to the property of being constant to image contrast and the identification of various types of features, the phase congruency model has been widely used in SAR image processing, such as image registration [7], target detection [32] and image segmentation [33]. However, in these applications, the phase congruency model is directly applied to the raw SAR image or the logarithm of the SAR image, which also suffers from speckle noise [34]. Inspired by the ratio-based edge detectors that provide the property of constant false alarm rate, we introduce the ratio-based method [35] and the monogenic signal [36] into the phase congruency to solve the aforementioned problem while maintaining a small computational cost.

The monogenic signal that was first proposed by Felsberg is the combination of a signal and its Riesz transform, which is computed as $f_{M}\left(x\right)=f\left(x\right)+f_{R}\left(x\right)$, where f(x) is the original signal, $f_{R}\left(x\right)=\left(h*f\right)\left(x\right)$ and h(x) is the spatial form of the Riesz transform. The Riesz transform, which is a two-dimensional generalization of the Hilbert transform, is defined as H(u)=iuiuuu, where $u=\left(u_{1},u_{2}\right)$. According to the definition of the monogenic signal, its energy is obtained as follows:

(4) $$\int |f_{M}{\left(x\right)|}^{2}\;dx\;=\int f{\left(x\right)}^{2}+{\left|f_{R}\left(x\right)\right|}^{2}\;dx\;=2\int f{\left(x\right)}^{2}\;dx,\;$$

which is two-times the energy of original signal, and it is only modified by a constant real factor. Consequently, the amplitude of the monogenic signal is isotropic, which means that there is no dependence on the orientation of the signal. Benefiting from the property of isotropy, we can calculate the local energy without orientations, which can reduce the computation cost. The Riesz transform can be regarded as two perpendicular Hilbert transforms, which correspond to two orthogonal filter banks. By substituting the isotropic model, the local energy can be derived as:

(5) $$\begin{array}{c} LE=\sqrt{f{\left(x,y\right)}^{2}+f_{R}{\left(x,y\right)}^{2}},\;\\ f_{R}\left(x,y\right)=\sqrt{{\left(\sum_{n}f_{h}\left(x,y\right)\right)}^{2}+{\left(\sum_{n}f_{v}\left(x,y\right)\right)}^{2}} \end{array}$$

(6) $$\begin{array}{c} f\left(x,y\right)=\sum_{n}I\left(x,y\right)*M_{n},\;\;\\ f_{h}=\sum_{n}I\left(x,y\right)*M_{n}^{h},\;\;f_{v}=\sum_{n}I\left(x,y\right)*M_{n}^{v}, \end{array}$$

where $M_{n}$ stands for an isotropic filter at scale *n* and $M_{n}^{h}$ and $M_{n}^{v}$ denote two orthogonal filters at scale *n*. As such, the amplitude can be derived as $A_{n}=\sqrt{{f_{n}}^{2}+{f_{h-n}}^{2}+{f_{v-n}}^{2}}$. Compared with the local energy model in Equation (2), the number of convolution operation has been reduced from 2no to 3n, resulting in a smaller computational cost.

**Figure 3:**
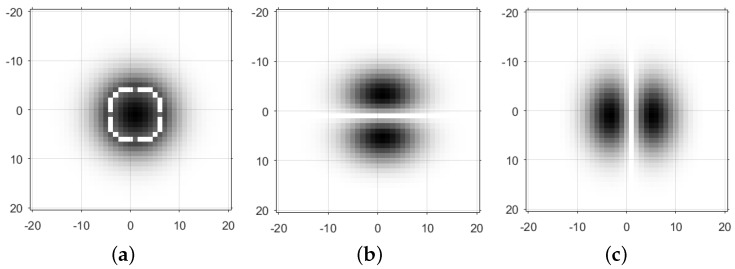
Three processing window. (**a**) Circle; (**b**) vertical; (**c**) horizontal.

However, the convolution of image and filter banks gives a poor performance for SAR images due to the multiplicative speckle noise. Hence, we replace the convolution with the ratio responses given by the filters. Similar to the aforementioned ROEWA operator, the ratio response is given by the ratio of local means of two non-overlapped sub-windows oriented at one angle [35]. Here, we choose the odd-symmetric part of the 2D Gabor filter to calculate the ratio responses, which is given as follows:

(7) $$G_{odd}\left(x,y\right)=\exp\left[-\frac{x^{2}+y^{2}}{2\sigma^{2}}\right]\sin\left[\omega (x\cos\theta +y\sin\theta )\right]\;,$$

where *w* represents the frequency of a sinusoidal wave, σ controls the scale of a Gaussian envelope and θ is the oriented angle. Figure 3b,c shows two odd-symmetric Gabor filters oriented in the vertical direction and the horizontal direction. The two filters correspond to $M_{n}^{h}$ and $M_{n}^{v}$, respectively. Consequently, the ratio responses can be computed as follows:

(8) $$f_{h}\left(x,y\right)=1-\min\left(\frac{\mu_{o}^{1}}{\mu_{o}^{2}},\frac{\mu_{o}^{2}}{\mu_{o}^{1}}\right),\;\;\;f_{v}\left(x,y\right)=1-\min\left(\frac{\mu_{o}^{3}}{\mu_{o}^{4}},\frac{\mu_{o}^{4}}{\mu_{o}^{3}}\right)$$

where $\mu_{h}^{1}$ and $\mu_{h}^{2}$ are local means of image intensity convolved with the horizontal processing windows, shown in Figure 3b, and $\mu_{v}^{1}$ and $\mu_{v}^{2}$ are local means of image intensity convolved with the vertical processing windows, shown in Figure 3c. Moreover, the convolution of image intensity with the isotropic filter $M_{n}$ can be also replaced with a ratio response, given as:

(9) $$f\left(x,y\right)=1-\min\left(\frac{\mu_{c}^{1}}{\mu_{c}^{2}}\right),$$

where $\mu_{c}^{1}$ and $\mu_{c}^{2}$ are local means of the two circle windows shown in Figure 3a. The scale parameter *n* relates to the size of Gauss templates, σ in the Gabor filter. Consequently, we can obtain ratio responses at different scales by adjusting the parameter σ. Substituting $f_{h}\left(x,y\right)$, $f_{v}\left(x,y\right)$ and f(x,y) into the local energy model, the improved SAR Phase Congruency model (named IS-PC) is derived by:

(10) $$IS-PC\left(x,y\right)=\frac{W\left(x,y\right)\left\lfloor \sqrt{f^{2}+{f_{h}}^{2}+f_{v}^{2}}-T\right\rfloor }{\sum_{n}\sqrt{{f_{n}}^{2}+{f_{h-n}}^{2}+{f_{v-n}}^{2}}+\varepsilon }.$$

We can see from the above equation, the number of convolution operations has been reduced from 2no to 3n. Benefiting from the robustness to speckle noise, our proposed IS-PC yields an accurate and integrated feature response while maintaining a small computational cost. The feature response obtained by the proposed IS-PC is utilized to further process in the next subsection. Comparative results on SAR images of the IS-PC with the conventional PC are presented in Section 5.3.

#### 3.2.2. Correspondence Detection by ISPCC and Selection Strategy

The computational cost of the traditional NCC operation is extremely high, especially for large remote sensing images. Lewis proposed a fast NCC [20], which consists of three main procures: First, the cross-correlation is calculated in the spatial or frequency domain depending on the image size. Then, local sums are calculated by precomputing running sums. The correlation coefficients are finally obtained by normalizing the cross-correlation using the local sums. Consequently, fast NCC of IS-PC (named ISPCC) is used as the similarity measure for fine registration, which is defined as:

(11) $$\begin{array}{c} ISPCC\left(u,v\right)=\frac{\sum_{\left(x,y\right)}\left[S\left(x,y\right)-\overset{-}{S_{u,v}}\right]\left[T\left(x-u,y-v\right)-\overset{-}{T}\right]}{{\left\{{\sum_{\left(x,y\right)}\left[S\left(x,y\right)-\overset{-}{S_{u,v}}\right]}^{2}\sum_{\left(x,y\right)}{\left[T\left(x-u,y-v\right)-\overset{-}{T}\right]}^{2}\right\}}^{0.5}} \end{array}$$

where *S* and *T* represent the IS-PC responses of the image and the template, $\overset{-}{T}$ is the mean of *T*, $\overset{-}{S_{u,v}}$ denotes the mean of *S* with the same size as the template and (u,v) is the coordinate of a point in image *I*.

**Figure 4:**
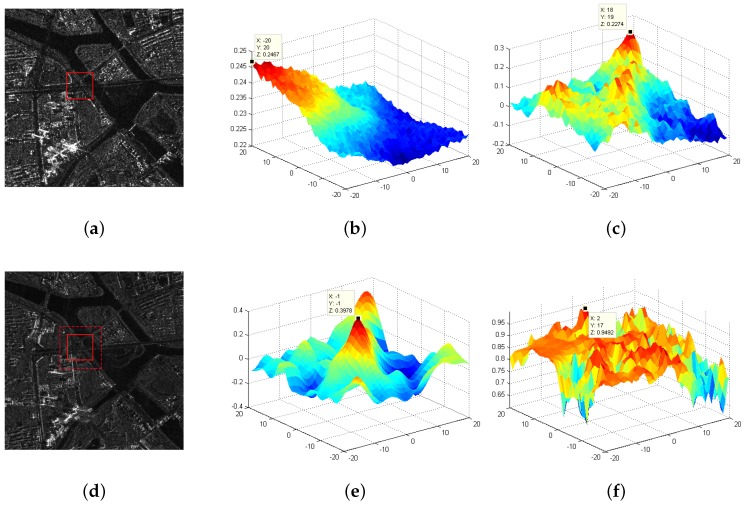
Comparison of the similarity curves. (**a**) The reference image; (**b**) MI; (**c**) fast NCC (fNCC); (**d**) the sensed image; (**e**) ISPCC; (**f**) LSCC.

In order to validate the matching performance of the proposed metric, we compare the ISPCC with the fast NCC (fNCC) of the image intensity, the MI of the image intensity and the NCC of local self similarity (LSCC) [8] by the similarity curves. A template-matching experiment is conducted on two correctly-matched SAR images, shown in Figure 4a,d. A template with a size of 51 × 51 is first selected in the reference image, then a local search region of [−20, 20], both in the x-axis and y-axis directions, is set in the coarse-registered image. The 2D similarity curves of four comparative methods are shown in Figure 4b–f, and the peaks are plotted in the curves, respectively. Since the two images have been correctly registered, the correct position is located at the center of the search region. We can see from the curves that only the ISPCC yields the correct result. For the fNCC method, the peak position is far from the center position. For the MI and LSCC methods, they detect several local extrema. Despite the MI and LSCC methods being able to handle nonlinear intensity differences, the test image is corrupted with speckle noise, and the intensity and geometry differences between the two templates are large, making the other three methods no longer suitable. Meanwhile, the running time of the fNCC, MI, LSCC and ISPCC is 0.1 s, 1.4 s, 2.19 s and 0.12 s, respectively (all the experiments were conducted with MATLAB R2014a). Consequently, the proposed ISPCC is not only robust, but also efficient, compared with the three other methods. It should be noted that the template size has a strong effect on the matching performance. More comparative analyses on the template size are presented in Section 5.3.

Uniformly-distributed Cps have been extracted in both the reference image and coarse-registered image. Templates are first selected for each Cp in the reference image, then corresponding points are obtained by finding the maximum in the local search region using the ISPCC measurement. Nevertheless, there still exist a few false correspondences that need to be eliminated. Here, we design a correspondence selection strategy, which consists of two steps: First, the matching quality of each correspondence can be measured by the result of ISPCC, where a large value corresponds to a good match. Ranking the matching qualities in descending order for each block, only the first half of the correspondences is selected as the candidates in this block. Second, the Fast Sample Consensus (FSC) proposed by Wu [37] is utilized to eliminate the misregistrations for all the remaining correspondences. The FSC first divides the candidates in RANSAC into two parts: the sample set that has a high correct rate and the consensus set that has a large number of correct matches, then an iterative method is put forward to increase the number of correct correspondences. Compared with RANSAC, FSC can get more correct matches using fewer iterations. Finally, the coarse-registered image can be rectified using an affine transform model that is estimated by the correct correspondences.

## 4. Experimental Results

In this section, in order to test the registration performance of the proposed algorithm, three pairs of GF-3 images are used. Details of the test images are listed in Table 1. Qualitative analyses of the registration accuracy and computational cost are demonstrated in Section 5, with comparative results of other state-of-the-art registration methods. These experiments were conducted with the MATLAB R2014a software on a computer with an Intel Core 3.2-GHz processor and 8.0 GB of physical memory.

### 4.1. Datasets

**Figure 5:**
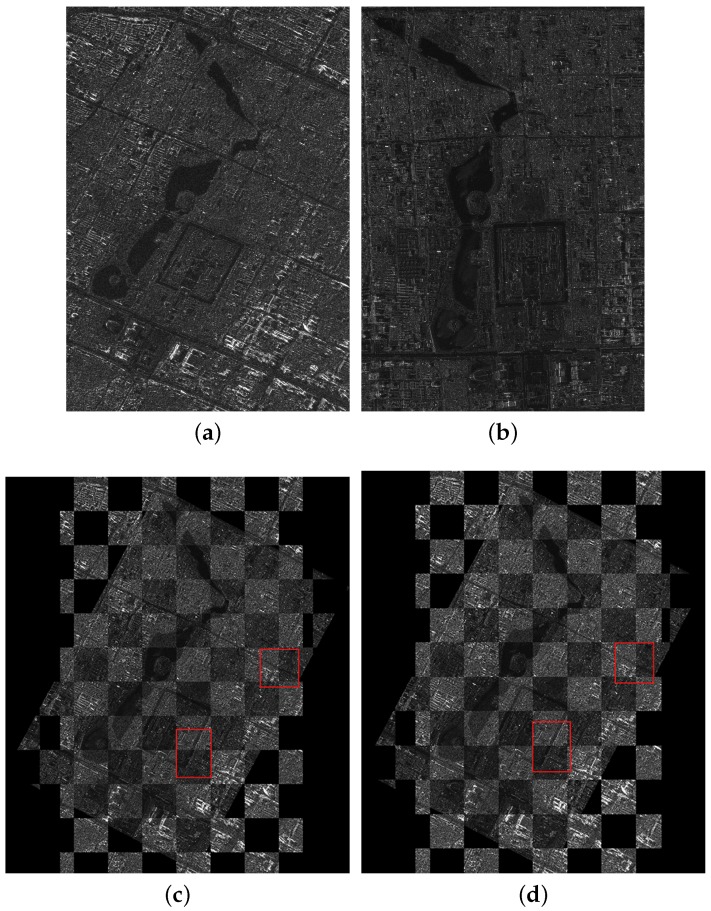
Registration results of the first image pair. (**a**) The reference image; (**b**) the sensed image; (**c**) the coarse registration result; (**d**) the fine registration result.

The first image pair consists of one SL image and one FSMI image describing the Forbidden City of Beijing, China. The resolutions of the two images are 1 and 5 m/pixel. We set the FSMI image as the reference image, which is shown in Figure 5a, and the SL image as the sensed image, which is shown in Figure 5b. The two images were imaged at different times and in different directions. The scale difference between them is five times, and we can observe that there exist a rotation difference (about 30°) and a translation difference. Since the two images have a large scale difference, we resize the two images to make a straightforward illustration, while the experiment still uses two images with different resolutions.

**Figure 6:**
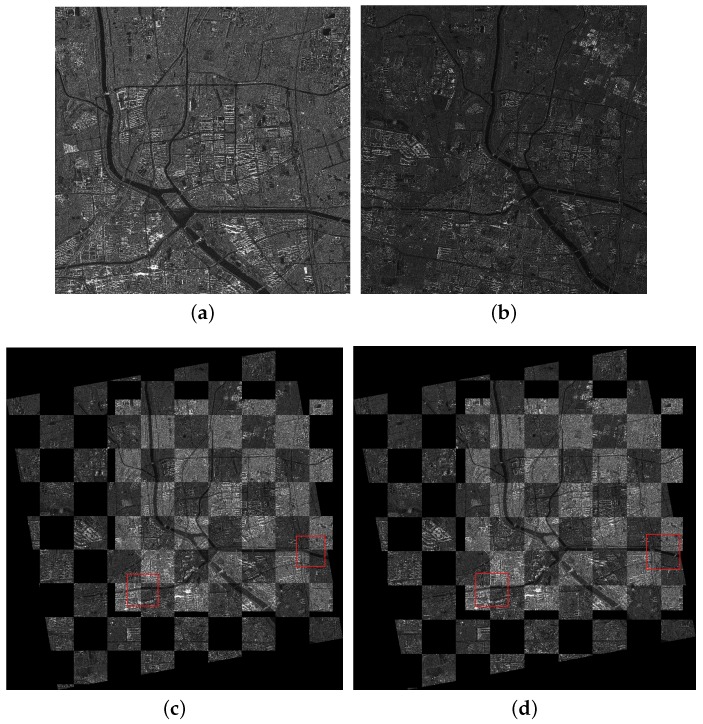
Registration results of the second image pair. (**a**) The reference image; (**b**) the sensed image; (**c**) the coarse registration result; (**d**) the fine registration result.

The second image pair consists of one UFSM image and one FSMI image describing a complex area with rivers and buildings in the east of Beijing City, China. The resolutions of the two images are 3 and 5 m/pixel. The FSMI image is set as the reference image, which is shown in Figure 6a, and the UFSM image is set as the sensed image, shown in Figure 6b. The two images have a scale difference (5/3 times), a rotation difference (about 10°) and a translation difference.

**Figure 7:**
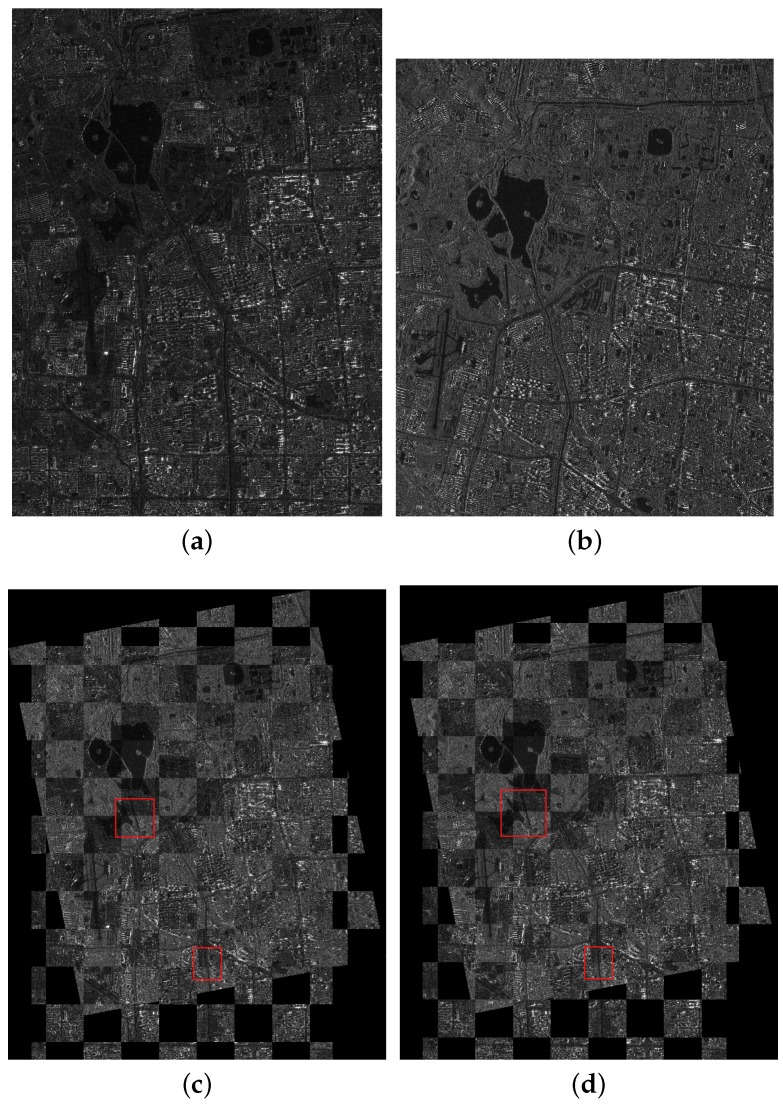
Registration results of the third image pair. (**a**) The reference image; (**b**) the sensed image; (**c**) the coarse registration result; (**d**) the fine registration result.

The third image pair consists of one FSMI image and one QPSMI image describing the Summer Palace and its surroundings in Beijing City, China. The resolutions of the two images are 5 and 8 m/pixel. The QPSMI image is set as the reference image, shown in Figure 7a, and the FSMI image is set as the sensed image, shown in Figure 7b. The two images have a scale difference (8/5 times) and a rotation difference (about 20°), and they were imaged at different times and in different directions.

### 4.2. Parameter Settings

In the coarse registration stage, the threshold $N_{t}$ that denotes the minimum number of remaining correspondences is set as six. The threshold −th used in the NNDR matching method is set to 0.9, and other parameters of SAR-SIFT follow the instructions in [9]. Parameters of the adaptive sampling method follow the instructions in Section 3.1. Since the SAR-SIFT algorithm has been proved to achieve a pixel-level Root Mean Square Error (RMSE) for multi-angle SAR images [9], we roughly set the localization deviation as five pixels for our test images from different imaging modes, then a local search region in the fine registration stage can be set as $\left[-5\cdot s,5\cdot s\right]$, both in the x-axis and y-axis directions, where *s* is the sampling times in the coarse registration stage. Both the reference and sensed image are partitioned into non-overlapped blocks with a size of 200 × 200, and *k* is set as 25, which refers to the number of Cps in each block. A larger number of *k* will improve the accuracy of fine registration, but the computational cost is also extremely increased. Consequently, k=25 is a reasonable choice. For the similarity metric ISPCC, four scales are used in the IS-PC detector. and other parameters remain the same as the phase congruency model. The template size is set to 51 × 51 pixels according to the template analysis in Section 5.3. For the selection strategy, half of the correspondences are first eliminated based on the ranking of matching quality, and the parameters of the FSC follow the instructions in [37].

### 4.3. Registration Results

**Figure 8:**
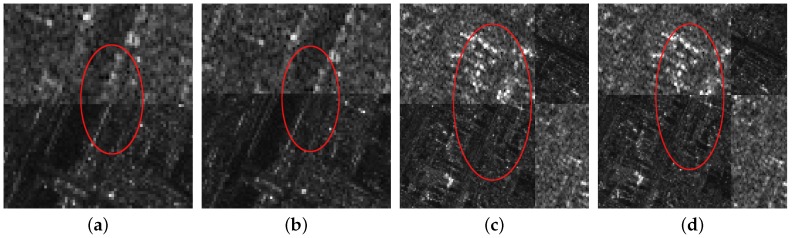
The enlarged subimages of the first image pair. (**a**) First subimage of coarse registration; (**b**) first subimage of fine registration; (**c**) second subimage of coarse registration; (**d**) second subimage of fine registration.

**Figure 9:**
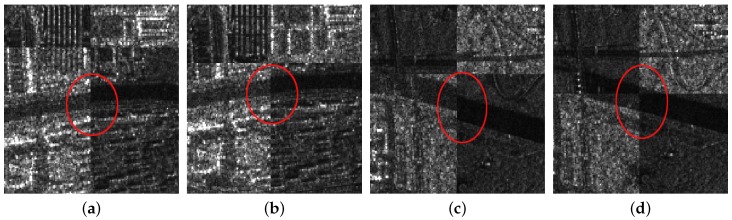
The enlarged subimages of the second image pair. (**a**) First subimage of coarse registration; (**b**) first subimage of fine registration; (**c**) second subimage of coarse registration; (**d**) second subimage of fine registration.

**Figure 10:**
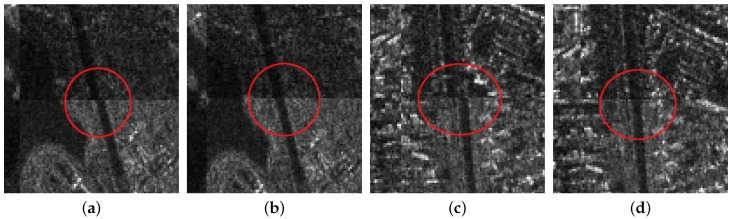
The enlarged subimages of the third image pair. (**a**) First subimage of coarse registration; (**b**) first subimage of fine registration; (**c**) second subimage of coarse registration; (**d**) second subimage of fine registration.

In the coarse registration stage, the AsSAR-SIFT algorithm (combination of the adaptive sampling method and the SAR-SIFT) is first used to eliminate the obvious scale, rotation and translation differences between the reference and the sensed images. Outliers are removed using FSC, and the transformation model is estimated by the correct corresponding keypoints between the images. Finally, the sensed image is rectified by the affine transform to form the coarse-registered image. Figure 5c, Figure 6c and Figure 7c illustrate the coarse registration results of the three image pairs, which are shown in checkerboard mosaicked images. In the fine registration stage, uniformly-distributed Cps are extracted using the feature responses calculated by IS-PC, then template matching is conducted by the ISPCC in a local search region for each Cp. Misregistrations are eliminated by a selection strategy. The coarse-registered image can be rectified using an affine transform model that is estimated by the correct correspondences. Figure 5d, Figure 6d and Figure 7d illustrate the fine registration results of the three image pairs. The enlarged sub-images of the coarse registration and the fine registration for all the image pairs are also presented in Figure 8, Figure 9 and Figure 10.

## 5. Discussion

In this section, the registration performance is evaluated in three ways. The first is the visual check by the checkerboard mosaicked image and the enlarged sub-images. The second measures are two quantitative criteria, RMSE and Correctly Matching Rate (CMR). The RMSE can be computed as:

(12) $$RMSE=\frac{1}{N_{corr}}\sum_{i=1}^{N_{corr}}{∥H\left(x_{1}^{i},y_{1}^{i}\right)-\left(x_{2}^{i},y_{2}^{i}\right)∥}_{2},$$

where *H* represents the ground truth transformation model between the reference image and the sensed image, ${∥\;\;∥}_{2}$ is the Euclidean distance and $\left(x_{1}^{i},y_{1}^{i}\right)$ and $\left(x_{2}^{i},y_{2}^{i}\right)$ are the coordinates of the *i*-th corresponding pair. The model *H* has been estimated between all three image pairs by manually selecting 20 pairs of corresponding control points for each image pair. While we use CMR in the comparative analysis of different template size, the CMR is defined as:

(13) CMR=NcorrNcorrNorigNorig,

where $N_{corr}$ is the number of correctly-matched Cps after eliminating false matches in the fine registration stage and $N_{orig}$ is the number of original Cps that are extracted in the first stage. The third is the computational cost. The running times of the three experiments are presented, both the coarse registration stage and the fine registration stage.

### 5.1. Discussion on the Registration Performance of Three Image Pairs

For the first image pair, since the size of the reference image is 810 × 1324, we set the sampling times as s=3. Operating the SAR-SIFT and AC-RANSAC methods on the sampled images, 22 pairs of corresponding points are extracted, which meets the requirement of the minimum correspondences. Then, the transformation model is estimated and the coarse registration is obtained by the model, shown in Figure 5c. From the enlarged sub-images shown in Figure 8a,c, we can see that there exists a deviation, such as misaligned edges. For the fine registration stage, we divide the reference and coarse-registered image into several 100 × 100 blocks, then Cps are extracted in each block. By using the ISPCC as the similarity metric, correspondences are obtained by searching a local region. The coarse-registered image is rectified by the correct correspondences. The enlarged subimages of the same region in the fine registration result are presented in Figure 8b,d. Compared with the subimages of the coarse registration result, the edges are precisely mosaicked.

For the second image pair, the size of the reference image is 1597 × 1554, so we set the sampling times as s=4. The remaining steps are the same as the first image pair. The coarse registration and fine registration results are shown in Figure 6c,d. It can be observed from Figure 9a,c that some misalignments still exist in the enlarged subimages. Corresponding subimages of the fine registration result are presented in Figure 9b,d, where the misalignments have been eliminated. For the third image pair, the size of the reference image is 958 × 1315, so we set the sampling times as s=3. The same conclusions can be obtained.

The RMSE and running time of both the coarse and fine registration stages for three image pairs are presented in Table 2. For SAR images with different viewpoints or different incidence angles, distortions often occur in mountains and urban areas (with high buildings). For mountain areas, they are very difficult to match due to the lack of keypoints. For urban areas containing high buildings, many keypoints can be detected in the building areas, and locations of these keypoints may have deviations due to the distortions. For the three image pairs in our experiments, the second image pair has the same viewpoints (DEC), resulting in the smallest RMSE. For the first and second image pairs, though the reference and sensed images have different viewpoints, building areas occupy only a small portion of the entire image. Moreover, in the fine registration, evenly-distributed control points reduce the influence of building areas, and the selection strategy can effectively remove outliers. Consequently, the registration accuracies of the three image pairs using our proposed technique are high. It can be observed that the running time of the coarse registration stage takes more than two-thirds of the total time. The computational complexity of the SAR-SIFT algorithm highly depends on the size of images. We have employed the adaptive sampling method to reduce its complexity. Moreover, all the experiments are conducted on the MATLAB software, and the computation efficiency can be further improved by implementing the proposed algorithm in C/C++.

### 5.2. Comparison with Other Registration Methods

**Figure 11:**
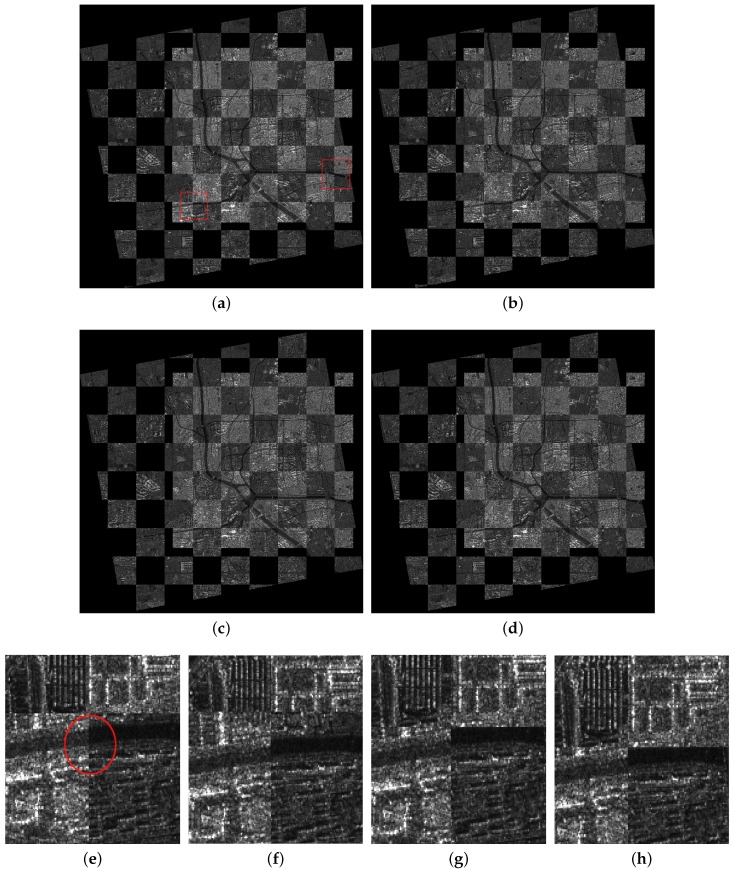

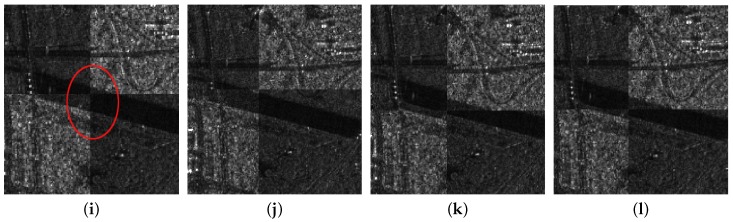
The comparative results of the second image pair. (**a**) The proposed method; (**b**) SAR-SIFT; (**c**) BFSIFT; (**d**) SIFT; (**e**–**h**) the first enlarged images of the proposed method, SAR-SIFT, BFSIFT, SIFT; (**i**–**l**) the second enlarged images.

The proposed algorithm has been compared with three registration methods, the original SIFT, SAR-SIFT and BFSIFT. The parameters follow their authors’ instructions. It should be noted that we use the AsSAR-SIFT algorithm in the coarse registration stage, which is a modified version of the SAR-SIFT algorithm. Comparisons of the RMSE and running time are presented in Table 2. We can see that the SAR-SIFT algorithm gives a good registration performance on the three SAR image pairs. Their RMSEs are 2.21, 1.89 and 2.46 pixels, respectively. Benefiting from the gradient by ratio, the SAR-SIFT descriptor shows a robustness to the speckle noise. However, the running time of SAR-SIFT on three image pairs is extremely long. Due to the large size of each image, the SAR-SIFT algorithm detects many keypoints in the image pyramid, and the descriptor construction for each keypoint is time consuming. For the AsSAR-SIFT used in the coarse registration stage, though its RMSEs are slightly larger than the SAR-SIFT algorithm, its running time has been highly reduced, such as a change from 900.2 s down to 18.71 s. For the BFSIFT and SIFT algorithms, their registration performances are poor on all three image pairs. Compared with SIFT, BFSIFT only replaces the Gaussian filter with the bilateral filter, so it will give better matching performance in regions with edge structures. Among the three image pairs, the second image pair that describes rivers and road has the largest number of edge structures. Accordingly, the registration performance of BFSIFT on the second image pair is the best among the three image pairs. Moreover, neither the SIFT nor the BFSIFT algorithm can overcome the problems of speckle noise and intensity differences between SAR images of different imaging modes. Comparative fusion results of the four algorithms are shown in Figure 11, along with two enlarged areas. From the enlarged subimages, we can see that the proposed method gives the highest matching accuracy, which is consistent with Table 2 and the previous conclusions. Overall, the proposed algorithm gives the smallest RMSE and shortest running time for all three image pairs. There are mainly three reasons, which are given as follows: (1) In the coarse registration stage, the AsSAR-SIFT method that integrates the SAR-SIFT algorithm and an adaptive sampling method decreases the computational cost while maintaining a fair registration performance. (2) In the fine registration stage, uniformly-distributed Cps are extracted, and the ISPCC used as a new similarity measure is accurate and efficient. (3) A selection strategy that combines matching quality ranking and the FSC method effectively removes outliers.

### 5.3. Comparative Analyses of the ISPCC

**Figure 12:**
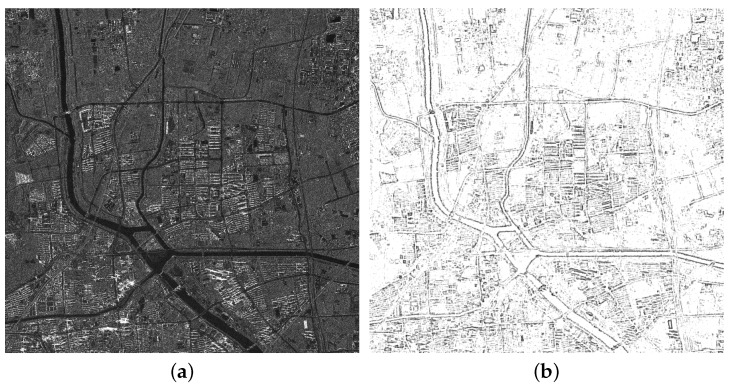

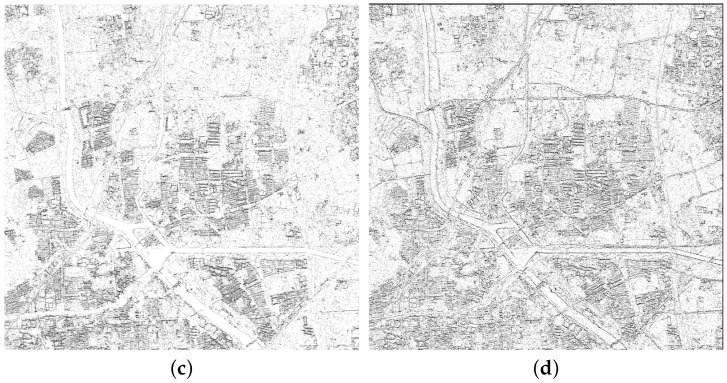
Detection results of three algorithms on the FSMI image. (**a**) The raw image; (**b**) IS-PC; (**c**) PC; (**d**) PC on the logarithm of the raw image.

The proposed ISPCC used as a new similarity measure is defined as the fast NCC of IS-PC. The IS-PC is proposed to calculate feature response of SAR imagery. In this section, we first evaluate the detection performance of IS-PC, compared with the conventional PC and PC on the Logarithm of the raw image (PCL). The FSMI image shown in Figure 6a is used to compare three detection algorithms. The detection results are shown in Figure 12, which are normalized to make an identical illustration. It can be observed that the proposed IS-PC gives a good feature response for this image. The edge structures of rivers and roads are precisely extracted, with good continuity and smoothness. The responses of homogenous areas are almost restrained. However, the PC method fails to give a reliable feature response due to the multiplicative speckle noise. The PCL yields a better result than the conventional PC. Even though the speckle noise is statistically well modeled as a stationary multiplicative process having negative-exponential first-order statistics and unity variance [38], the logarithm of speckle noise cannot be simply assumed as a Gaussian noise with a constant power spectrum. Therefore, the PCL algorithm is still affected by the speckle noise. Especially for the building areas, the structures extracted by the PC and PCL are strongly corrupted with noise. Moreover, the running times of IS-PC, PC and PCL are 1.52 s, 6.66 s and 6.66 s for the FSMI image with a size of 1554 × 1597, respectively. Consequently, the proposed IS-PC is robust and efficient to the extract feature response of SAR images.

**Figure 13:**
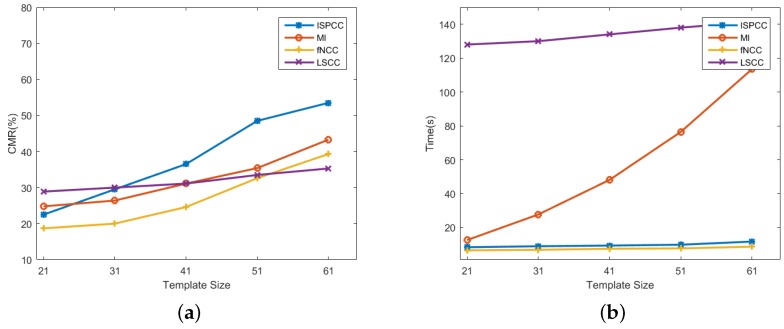
Comparisons of NCC, MI, LSCC and ISPCC on the second image pair. (**a**) Correctly Matching Rate (CMR) versus template size; (**b**) running time versus template size.

As mentioned in Section 3.2.2, the template size has a strong effect on matching performance. In order to analyze the sensibilities of ISPCC with respect to the template size, we then conduct a comparative experiment. The fine registration stage of the second image pair is selected to test the performance. Figure 13 shows the comparison results. The CMR is defined in Equation (13), where large CMR indicates that the matching result is more accurate and robust. It can be observed that the CMRs rise with the increase of template size for all the similarity metrics. A larger template size can contain more structural information, leading to an improvement on the distinctiveness of similarity metrics. The CMRs of ISPCC are larger than those of the other three metrics for sizes from 41 × 41–61 × 61; while the CMR of a size of 61 × 61 is nearly 2.5-times that of size 21 × 21. Moreover, the running time also rises with the increase of template size, since a large template size increases the computational cost. Benefiting from the advantages of IS-PC, the proposed similarity metric, ISPCC, is the most robust for SAR images. Considering the tradeoff of CMR and running time, the size of 51 × 51 is a proper choice for practical application.

## 6. Conclusions

Recently, GF-3, a multi-polarized C-band SAR satellite of the GF series that was launched in August 2016, has been widely used in scientific and commercial applications, such as disaster risk forecasting, water resources assessment and management, disaster weather, climate change forecast, and so on. Being the fundamental task of these image applications, SAR image registration needs to be accurate and efficient, which is still a challenging problem. Since GF-3 has the largest number of imaging modes among the SAR satellites in the world, we propose a robust and efficient registration algorithm for GF-3 images from different imaging modes in this paper.

The proposed approach consists of two stages, the coarse registration and the fine registration. First, an AsSAR-SIFT algorithm that integrates an adaptive sampling method and the SAR-SIFT algorithm is proposed to coarsely align two large SAR images efficiently. Then, obvious translation, rotation and scale differences are eliminated to further process. In the fine registration stage, the ISPCC that is defined as the fast NCC of an improved phase congruency model is used as a new similarity measure to find corresponding points in a local search region. The ISPCC is not only robust to speckle and intensity difference, but also discriminative and efficient. Moreover, uniform control points are extracted, and a selection strategy is used to remove outliers to achieve a stable and robust performance. In the experiments, three pairs of GF-3 images from different imaging modes are used to test the registration performance of the proposed algorithm. Experimental results show that the proposed algorithm improves the registration accuracy and decreases the computational cost, compared with other registration methods. However, there still exist some problems for our proposed algorithm. Though the proposed algorithm has good computational efficiency, it still cannot achieve real-time performance. The computational cost can be further reduced by implementing our algorithm in C/C++, which is one of our future works. Moreover, when the local distortion is extremely large, the registration performance may not be optimal. We think there exist three possible approaches to solve this problem: the first being to combine other additional information (e.g., using the building model and DEM) to rectify the location of keypoints; the second to select the on-ground keypoints for registration (e.g., intersections); the third to use a piecewise linear transformation model [39]. These possible approaches will be also done in our future work.

## Figures and Tables

**Table 1:** Image pairs and their characteristics. DEC is for the Descending direction, and ASC is for the Ascending direction.

Pair	Mode	Resolution	Date	Direction	Size
*a*	FSMI	5 m	16 August 2016	DEC	810 × 1324
*a*	SL	1 m	9 December 2016	ASC	3518 × 5387
*b*	FSMI	5 m	16 August 2016	DEC	1597 × 1554
*b*	UFSM	3 m	20 September 2016	DEC	3579 × 3611
*c*	QPSMI	8 m	9 December 2016	DEC	958 × 1315
*c*	FSMI	5 m	27 September 2016	ASC	1566 × 1896

**Table 2:** Comparison of SAR-SIFT, Bilateral Filter SIFT (BFSIFT) and the coarse and fine registration stages of the proposed method. ∗ denotes that the registration process has failed.

	Image Pair	SAR-SIFT	BFSIFT	SIFT	Coarse	Fine
RMSE (pixel)	a	2.21	4.48	5.88	3.64	0.605
Time (s)	a	900.2	654.1	709.2	18.71	9.32
RMSE (pixel)	b	1.89	3.56	4.5	4.64	0.465
Time (s)	b	3420.9	968.8	961.7	26.49	9.85
RMSE (pixel)	c	2.46	∗	∗	2.92	0.623
Time (s)	c	493.7	163.8	167.6	7.98	2.84
